# Supplementary material for: MetaQTL: a package of new computational methods for the meta-analysis of QTL mapping experiments
Source: BMC Bioinformatics. 2007 Feb 8;8:49. doi: 10.1186/1471-2105-8-49 (PMC1808479; doi:10.1186/1471-2105-8-49)
Supplement: Additional File 7 — MetaQTL Package : jar file and tutorial. This Zip archive contains both the MetaQTL JAR file and the files of the tutorial. [file 1471-2105-8-49-S7.zip › org.inra.metaqtl/doc/org/metaqtl/class-use/MetaChrom.html]

Uses of Class org.metaqtl.MetaChrom


|  |  |  |
| --- | --- | --- |
| **Overview** \| **Package** \| **Class** \| **Use** \| **Tree** \| **Deprecated** \| **Index** \| **Help** | |  |
| PREV   NEXT | **FRAMES**    **NO FRAMES**     **All Classes** |  |


---


## **Uses of Class org.metaqtl.MetaChrom**

| Packages that use MetaChrom | |
| --- | --- |
| **org.metaqtl** |  |
| **org.metaqtl.algo** |  |

Uses of MetaChrom in org.metaqtl

| Fields in org.metaqtl declared as MetaChrom | |
| --- | --- |
| `MetaChrom[]` | `MetaMap.chromosomes`             The meta chromosomes. |

Uses of MetaChrom in org.metaqtl.algo

| Methods in org.metaqtl.algo that return MetaChrom | |
| --- | --- |
| `protected  MetaChrom` | `MetaMapAlgorithm.buildMetaChrom(ChromCluster cluster)`             For a cluster of chromosomes `cluster` this method builds the consensus chromosome using a Weighted Least Square strategy and returns the result as a `MetaChrom` object. |

---


|  |  |  |
| --- | --- | --- |
| **Overview** \| **Package** \| **Class** \| **Use** \| **Tree** \| **Deprecated** \| **Index** \| **Help** | |  |
| PREV   NEXT | **FRAMES**    **NO FRAMES**     **All Classes** |  |


---
